# Syllabus of Lectures on Human Embryology

**Published:** 1895-01

**Authors:** 


SYLLABUS OF LECTURES ON HUMAN EMBRYOLOGY: AN INTRODUCTION TO THE STUDY OF OBSTETRICS AND GYNÆCOLOGY, FOR MEDICAL STUDENTS AND PRACTITIONERS, WITH A GLOSSARY OF EMBRYOLOGICAL TERMS. By Walter Porter Manton, M. D., Professor of Clinical Gynæcology and Lecturer on Obstetrics in the Detroit College of Medicine; Fellow of the Royal Microscopical Society, of the British Zoological, and American Microscopical Societies, &c., &c. Illustrated with Seventy Outline Drawings and Photo-Engravings. 12 mo. Cloth, 126 Pages. Interleaved for Adding Notes and Other Illustrations. $1.25 net. Philadelphia: The F. A. Davis Co., Publishers, 1914 and 1916 Cherry Street. 1894.

Except for students as an aid to note taking and for a rapid review of facts elsewhere gained, and for lecturers as an accurate and easy guide in the selection and outline of their subjects, this class of book has but little value. For the classroom this syllabus is well-arranged, and covers briefly the important subjects of anatomy of the female organs of generation; spermatozoon; spermatogenesis; the ovum, oogenesis and menstruation; general development of the embryo; uterine and fœtal membranes, placenta and utero-placental circulation; development of special organs and parts, heart, blood vessels and blood; general consideration of child at birth; changes in the maternal organism incident to pregnancy, practical work and a glossary of words and terms used in embryology. The book is splendidly illustrated with photo-engravings and outline drawings, with blank space around them for notes or drawings. Also, opposite each page in the book is a blank page for note taking. Embryology is a subject which students generally learn very little about and take very little interest in. The use of such a book as Prof. Manton's would greatly elucidate the subject, enable them to follow intelligently their lecturers and greatly aid their memories to retain a knowledge of this important introduction to obstetrics and gynæcology. V. H. T.

